# Impact of Environmental and Lifestyle Use of Chromium on Male Fertility: Focus on Antioxidant Activity and Oxidative Stress

**DOI:** 10.3390/antiox10091365

**Published:** 2021-08-27

**Authors:** Sara C. Pereira, Pedro F. Oliveira, Sónia Rodrigues Oliveira, Maria de Lourdes Pereira, Marco G. Alves

**Affiliations:** 1 Unit for Multidisciplinary Research in Biomedicine, Department of Anatomy, Clinical and Experimental Endocrinology, School of Medicine and Biomedical Sciences (ICBAS), University of Porto, 4050-313 Porto, Portugal; saracatarinapereira@gmail.com; 2 Química Orgânica, Produtos Naturais e Agroalimentares (QOPNA), Laboratório Associado para a Química Verde (LAQV), Department of Chemistry, University of Aveiro, 3810-193 Aveiro, Portugal; p.foliveira@ua.pt; 3 CICECO—Aveiro Institute of Materials, University of Aveiro, 3810-193 Aveiro, Portugal; sonia.oliveira@ua.pt (S.R.O.); mlourdespereira@ua.pt (M.d.L.P.); 4 Hunter Medical Research Institute, New Lambton, NSW 2305, Australia; 5 Department of Medical Sciences, University of Aveiro, 3810-193 Aveiro, Portugal

**Keywords:** trivalent chromium, hexavalent chromium, dietary supplements, antioxidants, male germ cells, fertility

## Abstract

Male reproductive tissues are strongly susceptible to several environmental and lifestyle stressors. In general, male reproductive health is highly sensitive to oxidative stress, which results in reversible and/or irreversible changes in testosterone-producing cells, spermatogenesis, and sperm quality. Chromium compounds are widely used in the +3 and +6 valence states, as food supplements, and in the industrial field, respectively. Chromium (III) compounds, i.e., Cr(III)-tris-picolinate, [Cr(pic)_3_], known as chromium picolinate, are used as nutritional supplements for the control of diabetes, body weight, and muscular growth. However, previous studies showed that animal models exposed to chromium picolinate experienced degenerative changes in spermatogenesis. Contradictory results are documented in the literature and deserve discussion. Furthermore, the long-term effects of chromium picolinate on the antioxidant system of treated subjects have not been properly studied. Comprehensive studies on the role of this compound will help to establish the safe and useful use of chromium supplementation. On the other hand, chromium (VI) compounds are widely used in several industries, despite being well-known environmental pollutants (i.e., welding fumes). Chromium (VI) is known for its deleterious effects on male reproductive health as toxic, carcinogenic, and mutagenic. Previous studies have demonstrated severe lesions to mouse spermatogenesis after exposure to chromium (VI). However, workers worldwide are still exposed to hexavalent chromium, particularly in electronics and military industries. Data from the literature pinpoints mechanisms of oxidative stress induced by chromium compounds in somatic and germ cells that lead to apoptosis, thus underlining the impairment of fertility potential. In this review, we analyze the benefits and risks of chromium compounds on male fertility, as well as the mechanisms underlying (in)fertility outcomes. Although supplements with antioxidant properties may maximize male fertility, adverse effects need to be investigated and discussed.

## 1. Introduction: Male Reproductive Health and Oxidative Stress at Brief

Oxidation–reduction reactions are an essential component of the intrinsic and complex communication network that keeps cells alive. Under normal conditions, there is a balance between antioxidant species and oxidant species to keep the reactive oxygen species (ROS) produced at several stages of the cell metabolism at normal physiological levels. However, several exogenous and endogenous sources of ROS can compromise this delicate balance, inducing oxidative stress (OS).

OS describes an imbalance between pro-oxidant (or oxidant) and antioxidant species in favor of oxidant species. Prolonged OS induces the oxidation of nucleic acids, carbohydrates, proteins, and lipids, leading to cell death [1]. Among the several ROS known to interfere with biological systems, the ones that most affect the male reproductive system are the hydroxyl radicals (OH) and the superoxide anion (O_2_^−^), along with hydrogen peroxide (H_2_O_2_). These species are naturally produced through the mitochondrial oxidative phosphorylation chain and other biological processes, accounting for 1–2% of the metabolized oxygen [2,3] in living cells. Testes are organs with naturally low oxygen tension and a very efficient antioxidant defense to protect both developing germ cells during spermatogenesis and Leydig cells, responsible for steroidogenesis. The two principal endogenous sources of ROS in the testicular tissue are testicular macrophages and defective sperm cells. Aziz N. and colleagues reported a positive association between specific sperm morphological defects and the production of ROS by sperm cells [4]. More specifically, these authors reported a positive correlation between sperm ROS production and sperm amorphous heads, damaged acrosomes, midpiece defects, cytoplasmatic droplets, and tail defects. However, the molecular mechanisms behind these correlations are yet to be unveiled. Regardless, Aziz N. and colleagues were able to establish a logistic regression analysis model that could identify individuals with low and high levels of sperm ROS production with an accuracy of 85%. This method could be very useful to predict the levels of ROS in the seminal plasma in facilities where methods to quantify ROS in samples are not available [4]. Gil-Guzman E and colleagues came to a similar conclusion, reporting that the retention of residual cytoplasm in sperm cells after spermiation is associated with excessive ROS production by the spermatozoa [5]. A molecular mechanism that could be involved in the production of ROS by defective sperm cells, specifically the ones with excess residual cytoplasm, could be due to the enhanced presence of glucose-6-phosphate dehydrogenase (G6PD). This enzyme catalyzes the regeneration of NADPH through the reaction with D-glucose-6-phosphate and NADP^+^. NADPH could be involved in the ROS generation, in a process mediated by the NADPH oxidase family [6,7].

Macrophages are associated with pro-inflammatory signals and can produce high quantities of ROS as a defense mechanism while releasing pro-inflammatory cytokines. Meanwhile, damaged spermatozoa are known to be a source of free electrons, which can promote ROS generation [8,9]. Both processes are important for the homeostasis of seminiferous tubules since each Sertoli cell is only able to sustain a limited amount of developing germ cells [10,11].

To maintain the balance between developing germ cells and Sertoli cells, a complex signaling pathway is responsible for selective germ-cell death. This process is also important to preserve the genomic integrity of the germline and eliminate irreparable defective cells [12]. Although the role of ROS in this process is still a debatable topic, authors propose that ROS may induce germ cell apoptosis through the activation of the p38 MAPK signaling pathway [13]. 

**Figure 1:**
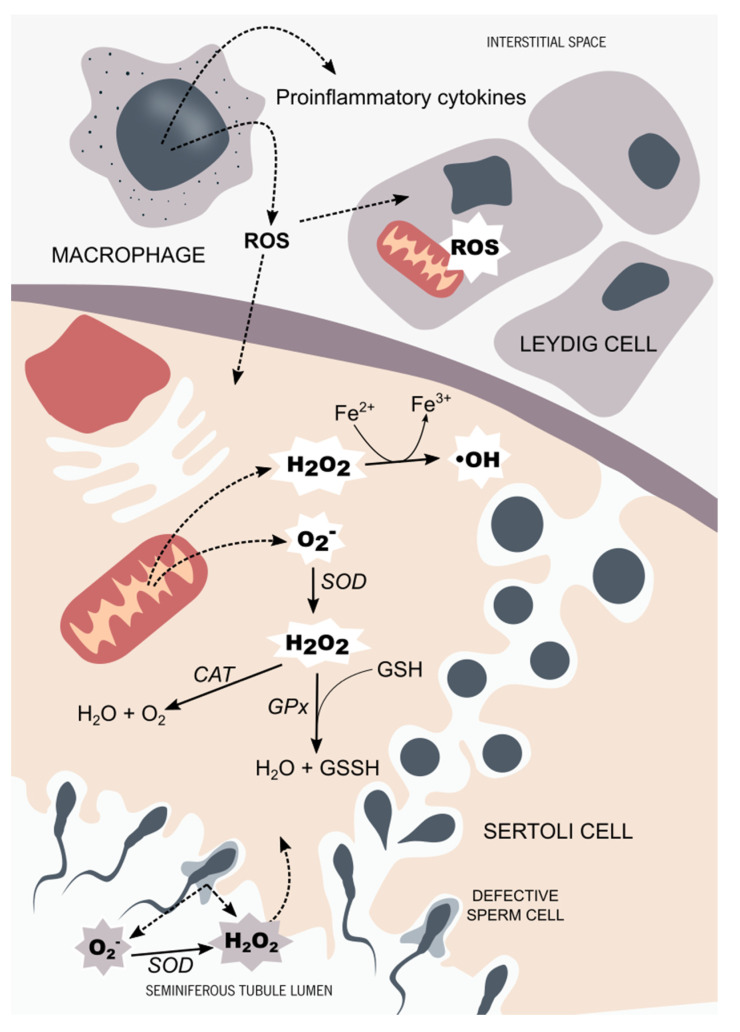
Testicular tissue oxidant/antioxidant balance. In the testicular tissue, the main sources of reactive species are activated in testicular macrophages and defective germ cells. The hydroxyl radicals (OH) and the superoxide anion (O_2_^−^), along with the hydrogen peroxide (H_2_O_2_), are the ROS that more often target the male reproductive system. Sertoli cells and Leydig cells are equipped with a very efficient antioxidant system. The main antioxidant enzymes found in the male reproductive system are superoxide dismutase (SOD), catalase (CAT), and glutathione peroxidase (GPx), along with non-enzymatic antioxidants, such as glutathione (GSH). Testicular cells are very sensitive to OS. Thus, a prolonged OS situation affects the steroidogenesis capacity of Leydig cells through mitochondrial impairment. Developing germ cells are also susceptible to OS, which is thought to be part of the complex signaling pathway responsible for selective germ-cell death. In order to maintain the homeostasis of seminiferous tubules, the oxidant, and antioxidant species balance each other, maintaining an adequate environment for steroidogenesis and spermatogenesis to occur.

Leydig cells can be found in groups (up to ten cells) adjacent to the seminiferous tubules, in the interstitial space of the testicular tissue. Steroidogenesis is mainly mediated by the pituitary gland through the luteinizing hormone (LH). In Leydig cells, LH activates the cyclic adenosine 3′,5′-monophosphate (cAMP) signaling pathway, promoting the mobilization of cholesterol into the mitochondria through the steroidogenic acute regulatory protein (StAR) [14]. The location of Leydig cells in the testicular tissue facilitates their interaction with the testicular macrophages. Inflammatory mediators, such as ROS and cytokines produced by activated macrophages, are known to interfere with steroidogenesis through the inhibition of the StAR protein expression [15]. Leydig cell aging is also intimately related to ROS, specifically ROS production associated with the P450 systems in steroidogenic cells [16]. This process is thought to be responsible for the age-associated decline in testosterone levels [17,18], although the mechanisms remain largely unknown (Figure 1).

After spermatogenesis, spermatozoa are stored in the epididymis. Herein, sperm cells pass through a maturation process, which comprises a series of membrane modifications and acquisition of surface proteins, among others. These events culminate with spermatozoa membrane and nuclear remodeling and motility acquisition. During the 10 days journey from the caput to the caudal region of the epididymis, sperm cells are prone to be exposed to OS, being defective sperm cells, macrophages, and activated B lymphocytes the main endogenous sources of ROS [19]. As in the testes, epididymis developed an efficient antioxidant system, in order to maintain ROS levels controlled during the transit and storage of spermatozoa. In the epididymis, glutathione peroxidases (GPx) and peroxiredoxins (PRDX) antioxidant families are the main mediators of the organ antioxidant defense, working cooperatively with each other, while being supported by a large variety of other antioxidant mediators, such as the superoxide dismutase (SOD), catalase (CAT), thioredoxins and thioredoxin reductase, and others (for review [20]).

Nevertheless, once in the female tract, low levels of ROS stimulate the cAMP pathway while promoting protein kinase A (PKA) activation. The activation of PKA subsequently leads to the activation of extracellular regulated kinase-like proteins and inactivation of tyrosine phosphatase activity [8,21]. All of these steps culminate in hyperactivation, a subcategory of capacitation, which refers to a specific state in which spermatozoa become highly motile [8]. The low ROS levels found in the female tract also promote the acrosome reaction and fertilization. After binding with the oocyte zona pellucida, the Ca^2+^ flux promotes the activation of the cAMP pathway and PKA [22]. ROS are also known to promote increased fluidity of spermatozoa membrane, a crucial event for successful fertilization [8,23]. 

Nevertheless, it is important to reinforce that the rise in ROS levels in both male and female reproductive tracts must be short-lived and controlled. Although essential for several molecular mechanisms regarding the development of germ cells, sperm capacitation, and fertilization, prolonged exposure to high ROS levels promote OS and consequently apoptosis. Aitken R.J. and colleagues were the first to propose that excessive amounts of ROS could have a severe impact on the fertilization capacity of sperm cells [24]. A previous study proposed that spermatozoa are susceptible to lipid peroxidation [25]. At this point, it was also reported that animal sperm were able to generate ROS, such as hydrogen peroxide [26,27]. Aitken R.J. and colleagues reported that the presence of calcium and a calcium ionophore (A23187) in the medium was able to boost the production of ROS by normal functional spermatozoa, levels peaking in only 5 min. Produced ROS were not originated in the mitochondria, since the addition of mitochondrial inhibitors was not able to prevent the rise of ROS. These authors demonstrated that sperm ROS production was inversely related to its oocyte-fusion capacity and proposed that the excessive activity of sperm ROS generating systems could be a cause of infertility [24]. In their subsquent work, the authors reported that membrane lipid peroxidation could be one of the main mechanisms by which ROS could impact the functional competence of the spermatozoa [28]. Nonetheless, the dangers of excessive ROS go beyond membrane lipid peroxidative, having the potential to promote severe DNA damage. ROS-induced mutations can occur at the testicular level, affecting the germline and the production of spermatozoa, as well as affecting the spermatozoa function and competence (for review [29]). Several studies have reported that infertile individuals have significantly more ROS in the ejaculate than healthy individuals [30,31,32,33,34]. After all these studies, a recent work [35] has proposed that OS could be a cause for male infertility. According to the most recent terminology, Male Oxidative Stress Infertility (MOSI) describes a condition in which high levels of ROS in the male tract impact the fertility potential of individuals [35]. It is assumed that up to 80% of infertile males have elevated levels of seminal ROS, increasing sperm DNA damage, and lowering the chances of fertilization and/or development of healthy embryos [35].

The delicate balance between oxidant and antioxidant species is not only affected by endogenous sources. With industrialization, humans and all forms of life have become exposed to a new panoply of chemical compounds. Heavy metals, such as lead, chromium, cadmium, zinc, and nickel are commonly used in several industries, mainly for their anticorrosion proprieties. However, these compounds are highly polluting and can contaminate water supplies, air, and soil. This way, compounds encounter all kinds of living creatures, thereby entering the food chain. It is suggested that OS is one of the main mechanisms by which metallic compounds can promote their hazard effects on health [36,37]. Since the reproductive system of both males and females is highly susceptible to oxidative damage, it is also proposed that exposure to these metallic pollutants may induce infertility. Chromium (VI) is popularly used in the metal and paint industries for its anticorrosion properties. This oxidation state is known to be mutagenic and carcinogenic and is often found in industrialized areas [38]. However, chromium (III) is considered an essential element and is often found in dietary supplements especially recommended for diabetic individuals [39]. Withal, its impact on the reproductive potential of individuals is largely unknown. Herein, we review the existing literature regarding the impact of chromium (VI) and (III) on the male reproductive system and its impact on the oxidative balance of the testicular tissue, seminal fluid, and spermatozoa. 

## 2. Chromium: Biochemistry and Derivates

Chromium was discovered by the French chemist Nicolas-Louis Vauquelin in 1797. He was able to isolate the metal by boiling a mixture of chromic acid and carbon in a graphite crucible, leading to the precipitation of a metal, which he called chromium, from the Greek word *chrōmos*, which means color. In the years that followed, several other chemists were able to isolate the new metal [40,41]. 

This element can be abundantly found in the environment, although never as a pure metal. Due to its high resistance to corrosion and oxidation, chromium is commonly added to other metals, such as iron, nickel, steel, and several others, playing a crucial role in the development of new technologies [41]. 

On the periodic table, chromium is the first element of group 6, period 4, placing it in the transition metal group. It has an atomic number 24, with six valence electrons distributed in the 3*d* and 4*s* orbitals. The electronic configuration of chromium is usually represented as:

(1) $$\left[Ar\right]\;3d^{5},\;4s^{1}$$

This unusual electron configuration relies on the fact that half-full *d* orbitals are more stable than full *s* orbitals. By transferring an electron from the 4*s* orbital to the 3*d* orbital, chromium gains extra stability. This also means that chromium is very prone to oxidation. Of the six possible oxidation states, the most common are +3, and +6, also known as chromium (III) and chromium (VI). 

Chromium (III) has been highly studied due to its biochemical properties. In the 1950s, researchers W. Mertz and K. Schwarz started to unveil the role of chromium biochemical properties, demonstrating that factor 3 (as the authors referred) was able to prevent liver necrosis and glucose intolerance in rats fed with a *Torula* yeast-based diet [42]. In subsequent years, the authors proposed that a biologically active form of chromium (III) was part of a complex, the glucose tolerance factor (GTF), which could participate in glucose metabolism [43]. Although the role of GTF was extensively studied in the years that followed, its structure and molecular mechanism of action remained unresolved for decades. At the end of the 20th century, some works started to question the existence of the chromium complex. The work of Hwang D. and colleagues [44] and of M. Simonoff and colleagues [45] both supported this hypothesis. The pair of studies used Cr-rich yeast fractions and evaluated chromium biological effects on glucose oxidation in rat adipocytes. Despite the different methodologies used by the two groups, the authors failed to correlate the chromium content of the yeast fractions to the GTF-activity (evaluated by the glucose oxidation on rat adipocytes). Both studies supported that the chromium–GTF complex was actually an artifact of the isolation method and did not exist, further supporting that chromium was irrelevant for glucose metabolism [44,45]. At this point, chromium had been considered an essential element for over 30 years and the National Research Council (U.S.) had estimated that the daily dietary intake of chromium should be set at 50–200 mg per day [46]. Furthermore, the intake of chromium supplements, more specifically, chromium picolinate [Cr(pic)_3_], was already popular. Nowadays, the biochemical role of chromium (III) is controversial, and some suggest that chromium should actually be removed from the list of essential elements [39,47]. 

Although the effects of chromium (III) have just started to be unveiled, the carcinogenic and mutagenic effects of chromium (VI) have been known for a long time. Chromium (VI) is human-produced through the oxidation of chromium (III). As a powerful oxidant, chromium (VI) is very commonly used for anticorrosion and preservation of metals. It is also used as an anticorrosive agent in paints and primers. 

Due to its high solubility, chromium (VI) can easily contaminate water supplies and the air, making it highly pollutant [38]. Chromium (VI) is usually found in the form of chromate oxyanion: [CrO_4_]^2−^. This ionic form is very similar to the sulfate oxyanion: [SO_4_]^2^. Its similar structure allows chromium (VI) to easily enter the living cell through anionic exchange channels [48]. Once in the living cell, chromium (VI) undergoes a series of reduction reactions to achieve its much more stable form of chromium (III). This process induces the generation of genotoxic intermediates, being responsible for the mutagenic and carcinogenic effects of chromium (VI).

## 3. Chromium Picolinate Applications and Mechanisms of Action

The publication of W. Mertz and K. Schwarz’s work, in 1955 [42], opened doors to the development and commercialization of chromium (III) dietary supplements. In the 1960s, studies reported that the effects of chromium on glucose metabolism were dependent on insulin [49,50]. Shortly thereafter, oral supplementation of chromium (III) was suggested to improve glucose tolerance in diabetic rats [51]. The improvement of glucose tolerance after oral chromium (III) supplementation has also been reported in human adults and children [52,53,54]. All these studies defended that chromium was essential for carbohydrate and lipid metabolism, being particularly beneficial for glucose-intolerant individuals. This evidence promoted the popularization of chromium supplements in the following decades. 

Chromium picolinate was first described in 1917 but gained popularity as a dietary supplement in the late 20th century due to its ability to promote body fat loss and increase lean muscle mass, along with increased insulin internalization and glucose uptake [55,56]. Furthermore, chromium picolinate had a higher absorption rate (~2%) when compared to other dietary chromium complexes (~0.5%) [57].

**Figure 2:**
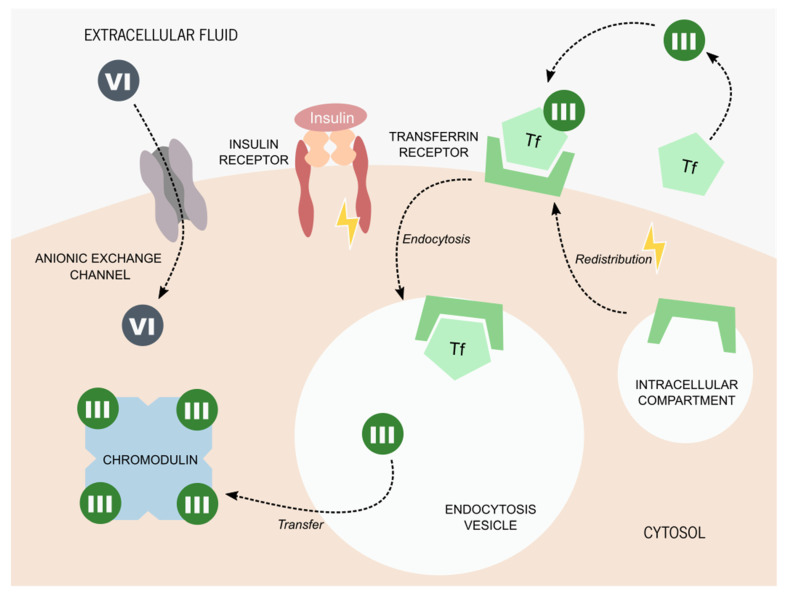
Proposed molecular mechanisms for chromium internalization. Chromium (VI) is usually found in the form of chromate oxyanion: [CrO4]^2−^, which is part of the chromate compounds. Its similar ionic structure to some essential anions allows chromium (VI) to enter living cells through its anionic exchange channels. The process of chromium (III) internalization is still a matter of debate. The most accepted hypothesis states that chromium (III) is captured by serum proteins, such as transferrin (Tf). The presence of insulin promotes the redistribution of the Tf receptor from intercellular compartments to the cytoplasmatic membrane. Tf bound to chromium (III) is captured by the Tf receptor and internalized by endocytosis. Once inside the cell, chromium (III) is transferred to chromodulin, an oligopeptide capable of binding to four equivalents of chromium ions. It is thought that chromodulin can enhance insulin effects on glucose and lipid metabolism.

The mechanism of chromium (III) uptake by living cells is still a matter of study, even after all these years. Studies proposed that chromium (III) could be carried by serum proteins, such as transferrin [58,59]. This protein is the main mediator of iron transport, although it can also transport other transition metals, including chromium [60]. Transferrin is captured from the serum into the cells through endocytosis, in an insulin-dependent-process [61]. Insulin is known to promote the redistribution of the transferrin-receptor (TfR) from an intercellular membrane compartment into the cytoplasmic membrane. Herein, it captures the iron-transporting transferrin from the serum, inducing a cascade of molecular events that culminate in the endocytosis of TfR coupled to transferrin [61]. This process has been described in adipocytes [62], hepatocytes [63], and glioma cells [64]. It is proposed that chromium (III) can enter living cells through a similar process, being captured by transferrin, or another metal transporting serum protein, and being captured by its membrane receptors [65]. Once inside the cells, chromium (III) is transferred to an oligopeptide known as low-molecular-weight chromium-binding substance (LMWCr), also known as chromodulin [66]. This complex was firstly described by Yamamoto A. and colleagues, who proposed the existence of an anionic complex with a tetranuclear assembly, capable of binding to four equivalents of chromium ions [66]. This complex is ubiquitous in mammals and it appears to amplify the effects of insulin on glucose and lipid metabolism through the stimulation of protein tyrosine kinases (which include the insulin receptor), phosphotyrosine phosphatases, and other enzymes [51,67]. Nevertheless, it has also been hypothesized that the LMWCr function resides mainly in the capture and excretion of chromium [68]. This would also justify the wide distribution of this complex in different cell types and mammals [68] and its rapid elimination through the urine after the ingestion of glucose (a phenomenon reported by several authors [69,70,71]). Regardless, chromium picolinate appears to have, in fact, beneficial antidiabetic effects by potentiating the insulin action [72,73,74,75] (Figure 2).

Since insulin can also promote the endogenous synthesis of fatty acids and triglycerides, as well as promote muscle protein synthesis, it is thought that chromium picolinate can promote these processes by boosting insulin effects. Over the years, studies have proposed that chromium picolinate helped to improve body composition, promoting the maintenance (or increase) of lean body mass while enhancing the loss of fat mass [76,77]. These characteristics were particularly appealing for athletes [78,79,80,81]. However, several of the studies involving athletes on diets supplemented with chromium picolinate failed to report significant improvements in the body composition and strength of the athletes [80,81,82]. 

Due to this ambiguity on the effects of chromium picolinate supplementation, researchers soon started to question the safety of this complex. The work of Stearns D.M. and colleagues reported that chromium picolinate promoted chromosome cleavage and mutations in Chinese hamster ovary cells [83,84]. The same group also reported that this complex could promote apoptosis and mitochondrial damage [85]. However, the concentrations of chromium picolinate used in these studies (0.05–1 mM) were much higher than the physiological concentration of chromium reported in individuals on diets supplemented with chromium picolinate [80,81,82]. In fact, the concentration of chromium picolinate found in human liver cells after 5 years of supplementation was as high as 13 µM [86]. Fortunately, according to a study by Anderson R.A. and colleagues, chromium picolinate supplementation (100 mg of Cr per kg of diet) of four-week-old rats for 24 weeks had no toxic effects. Nonetheless, the authors noticed that the chromium concentration in the liver and kidneys of the animals increased linearly [87]. The amount of chromium picolinate used in this study were equivalent to a chromium supplementation of 750 mg per day to a 50 kg human, a value 1000-fold higher than the concentration of chromium picolinate often used in human diet supplementation [88]. In summary, although the beneficial effects of dietary supplementation with chromium picolinate are still debatable, it appears that no harmful effects are associated.

## 4. Chromium Compounds and Reproductive Health

Chromium is a very common element in the earth’s crust, which means that it can be found naturally in all types of ecosystems. However, human industrial activities have promoted environmental contamination with harmful chromates, the most common form of chromium (VI). Due to its high solubility, this pollutant can contaminate water supplies, air, and soil, impacting ecosystems and forms of life [38]. In plants, chromium (VI) is known to alter the germination process of seeds and the growth of roots, stems, and leaves [89]. Furthermore, chromium also disturbs photosynthesis and promotes several other metabolic deleterious effects [89]. The impact of chromium (VI) in mammals, specifically in the reproductive system, has also been explored in recent years. Aruldhas M. and colleagues reported that chromium (VI) is extremely toxic for testis [90]. In that study, Bonnet monkeys were exposed to chromium (VI) (100, 200, and 400 p.p.m.) through their drinking water for 6 months. The treatment promoted the complete disruption of spermatogenesis, with the premature release of germ cells (in several stages of development) into the lumen of seminiferous tubules. Spermatocytes had fragmented chromatin, swollen mitochondria, and vacuolation. Moreover, the presence of macrophages with phagocyted sperm cells suggested the disruption of the blood–testis barrier. The authors suggested that chromium (VI) could disrupt spermatogenesis by inducing free radical toxicity [90]. The analysis of the chromium (VI)-treated animals’ epididymis through transmission electron microscopy revealed an increased abundance of basal cells and intraepithelial macrophages and increased cytoplasm in both cell types [91]. The higher electron density of macrophages suggested phagocytosis of sperm cells and other cellular debris. The cytoarchitecture of principal cells was normal in chromium (VI)-treated animals, and its vacuolated appearance suggested endocytosis activity. The authors observed spermatozoa in process of disintegration inside the vacuoles of several principal cells. The authors proposed that the increase in pathophysiological spermatozoa, due to chromium (VI) disruption of spermatogenesis, resulted in the increase of phagocytic activity by principal cells [91]. Since these cells do not have the mechanisms necessary to process high quantities of lipofusion material, which results from the disintegration of dead/damaged spermatozoa, these residues are discharged to the intracellular space and taken by the basal cells and intraepithelial macrophages, leading to the accumulation of these cells in the epididymal epithelium and promoting ductal obstruction [91,92]. 

In the study by Marouani N. and colleagues, male Wistar rats were injected with potassium dichromate (1 and 2 mg/kg) [93]. The authors evaluated several markers for OS in the testis of the treated animals. The authors reported that 15 days after treatment, increased lipid peroxidation and metallothionein levels were found in the testicular tissue of these animals, while CAT activity was decreased. The treatment also promoted an increase in DNA degradation in the testicular tissue, which ultimately resulted in germ cell apoptosis [93]. In another study, pregnant Sprague Dawley rats were given 0, 3, 6, and 12 mg/Kg of chromium (VI) in the form of potassium dichromate [94]. The testes were collected from the pups and the distribution, number, and function of Sertoli and Leydig cells were investigated. The authors reported that a dose of 3 mg/Kg of chromium (VI) induced the upregulation of testosterone production, probably through the increased expression of the Luteinizing hormone/choriogonadotropin receptor (Lhcgr) gene. The StAR mRNA and protein levels were not affected by the 3 mg/Kg chromium (VI) treatment. The proliferation of Leydig cells was also unaffected. Interestingly, the 12 mg/Kg dose of chromium (VI) had devastating effects on the pups testis physiology: the percentage of Leydig cell population increased and cell growth was retarded. The expression of the Lhcgr was downregulated, as well as the StAR expression, resulting in decreased levels of testosterone. This study proposed that chromium (VI) appears to have biphasic effects on rat fetal Leydig cells, being able to promote or downregulate Leydig cell maturity and steroidogenesis in a dose-dependent manner [94]. 

Hong L. and colleagues explored how exposure to chromium (VI) could impact the fertility potential of men [95]. They followed a group of 21 workers exposed to chromium (VI) from an electroplating facility. This group of men had significantly lower sperm counts, with significantly lower motility, compared to the non-exposed group. The chromium (VI)-exposed group also had higher levels of serum follicle-stimulating hormone (FSH), which could be related to low sperm counts. Meanwhile, the exposed group also presented a decreased zinc concentration in the seminal plasma, suggesting a decrease in the overall antioxidant potential. Altogether, the authors proposed that men exposed to chromium (VI) had a decreased fertility potential compared to unexposed men [95]. The same conclusions were reached by other authors who also followed the fertility potential of chromium (VI)-exposed men [96,97]. Yang Y. and colleagues reported that female workers from factories that exceeded chromium hygienic standards had an increased risk of abortion (2.13-fold) and threatened abortion (20.17-fold) than female workers from other industries [98]. Hjollund N. and colleagues reached similar conclusions when studying pregnant women whose partners were engaged in the stainless-steel welding industry, which is associated with the inhalation of chromium (VI) [99]. The authors reported that these women had an increased risk of abortion, demonstrating that paternal exposer to chromium (VI) could impact the outcome of pregnancy despite mothers not being exposed [99].

Contrarily to chromium (VI), the impact of chromium (III) in the reproductive system was disregarded for several decades. To test if chromium supplementation in males could also impact the offspring’s health, McAdory A. and colleagues supplemented male CD-1 mice with 200 mg/Kg chromium picolinate for 4 weeks before mating [100]. Each male was mated with two females. After mating, females were individually housed and fed with standard rodent chow and water ad libitum. No difference was found in the litter size of males supplemented with chromium picolinate or non-supplemented (Control). The authors reported an increase in the average number of total resorbed or dead fetuses in the supplemented group when compared to the control. The fetuses had also a tendency to weigh significantly more in the treated group than in the control group, although none of the reported differences were statistically significant. The authors concluded that, in mammals, it is unlikely for chromium picolinate to induce severe harmful effects to the offspring of males supplemented with reasonable doses of this compound [100]. 

Regarding the impact of chromium (III) supplementation in the physiology and function of the testis, several contradicting studies have been published. Ferreira M. and colleagues treated male adult CD1 mice with 25 and 50 mg/kg (of body weight) of chromium picolinate daily for two weeks [101]. Testes were collected, and a histological study was performed. The authors reported that considerable damage was present in the testis of mice of both chromium-treated groups. Degeneration of the seminiferous tubules epithelium, vacuolation, and sloughing of immature germ cells were some of the damage types presented. Further, the group treated with the highest dose of chromium picolinate (50 mg/kg) also presented strong atrophy of the seminiferous tubules, with vacuolation and absence of germ cells [101]. Meanwhile, Dallago and colleagues supplemented 14-week-old Santa Inês male lambs with chromium picolinate (0.8, 9.0, 12.0, and 21.0 µg/kg of body mass) daily for 84 days. No morphological alterations were found in the testis of these animals, and the seminiferous tubules presented a normal conformation [102]. Horký P. and colleagues supplemented breeding boars with chromium picolinate (181.81 µg/kg of feed ration) daily for 95 days [103]. Sperm analysis was performed at four different time points (days 18, 49, 77, and 95). No significant differences were found regarding sperm quality between the chromium-treated and non-treated groups, although a tendency was found for the percentage of pathological sperm to be lower in the chromium-treated group at days 77 and 95 [103]. Similar results were found by Shanmugam M. and colleagues, where Dahlem Red peripubertal roosters were supplemented with organic chromium (yeast chrome) (0.3, 0.6, and 0.9 mg/kg diet), daily for three months. The authors concluded that chromium supplementation of peripubertal roosters did not affect semen quality nor the fertility potential of animals [104]. In contrast, other avian studies have reported an improvement in sperm quality in animals after chromium supplementation [105,106]. In sum, several contradictory conclusions have been reported on the impact of chromium (III) supplementation on male fertility potential, most likely due to the wide variety of different methodologies used between studies. The impact of chromium (III) supplementation on the fertility potential of men remains to be uncovered.

## 5. Redox Balance in Male Reproductive Tissues and Chromium Compounds

In the male reproductive system, OS promotes devasting effects on the fertility potential of individuals. Some of the pathological effects of OS on the testicular tissue are the lipid peroxidation of spermatozoa cytoplasmatic membranes (rich in polyunsaturated fatty acids), sperm DNA fragmentation, and steroidogenesis impairment, among others [8]. 

The role of chromium (III) in the regulation of OS in the testis remains to be uncovered. Nonetheless, some studies on the impact of chromium (III) supplementation on the male fertility potential have also investigated the presence of OS biomarkers. In a study by Shanmugan M. and colleagues, Dahlem Red peripubertal roosters were supplemented with organic chromium (yeast chrome) (0.3, 0.6, and 0.9 mg/kg diet), daily for three months. The authors investigated lipid peroxidation of seminal plasma through malondialdehyde (MDA) quantification. However, seminal plasma lipid peroxidation was not affected by chromium supplementation [104]. Meanwhile, Biswas A. and colleagues reported an increase in MDA levels in the seminal plasma of male turkeys supplemented with a 500 and 750 µg/kg diet of chromium picolinate [105]. This rise in MDA could suggest an increase in lipid peroxidation levels in the seminal plasma of chromium-treated animals. However, the effects of chromium (III) on the oxidative potential of male reproductive tissues remain elusive.

**Figure 3:**
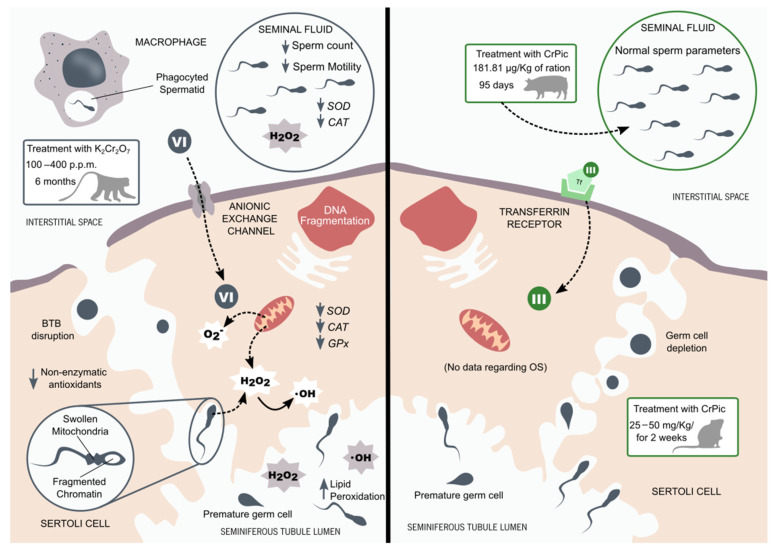
Chromium effects in males’ reproductive system. The Aruldhas M. and colleagues’ studies with Bonnet monkeys treated with potassium dichromate (K_2_Cr_2_O_7_) reported a complete disruption of spermatogenesis and blood–testis barrier (BTB). Spermatids had fragmented chromatin and swollen mitochondria. The activity of testicular antioxidant enzymes was decreased, along with the concentration of non-enzymatic antioxidants. Meanwhile, levels of hydrogen peroxide (H_2_O_2_) and hydroxyl radical (·OH) increased with the chromium (VI) treatment. The authors also reported a decrease in SOD and CAT activities in both sperm and seminal plasma. Decreased sperm count and motility could be found in the semen of treated animals [90,107]. It is suggested that chromium (III) promotes its deleterious effects through the induction of oxidative stress (OS) in the testicular tissue. The treatment of CD1 mice with chromium picolinate (CrPic) by Ferreira M. and colleagues reveals degeneration of the seminiferous tubules, depletion of germ cells, and sloughing of immature germ cells [101]. However, the supplementation of breeding boars with CrPic performed by Horký P. and colleagues reveals no alterations in the semen quality of these animals [103]. No data have been found on the impact of chromium (III) on the oxidative balance of the testicular tissue.

While the antioxidant/oxidant effects of chromium (III) remain a topic of debate, no doubts are left regarding the devasting oxidative damage that chromium (VI) may induce in the testicular tissue. Along with hazard effects of chromium (VI) in the physiology and function of Bonnet monkeys’ testis described by Aruldhas M. and colleagues, the authors also investigated the activity of antioxidant enzymes and the abundance of non-enzymatic antioxidants [90]. The activity of testicular SOD, CAT, and GPx was decreased in monkeys treated with chromium (VI) in a dose-dependent manner. The activity of testicular γ-Glutamyl transpeptidase (γ-GT) only decreased in the group treated with the higher dosage of chromium (VI) (400 p.p.m.), and the activity of testicular glutathione-S-transferase (GST) increased in all treated groups (100, 200, and 400 p.p.m.). The treatment with chromium (VI) also induced a decrease in the concentration of vitamins A, C, and E and an increase in the concentration of reduced glutathione. While the concentration of antioxidants decreased, in general, with chromium (VI) treatment, the concentration of hydrogen peroxide and hydroxyl radicals increased with the treatment in a dose-dependent manner. This led to the conclusion that chromium-induced histological damage in primates was promoted by the increased OS in the testis [90]. The same group also evaluated the effects of chromium (VI) treatment on the semen of treated monkeys [107]. The authors reported that chromium (VI) treatment induced a decrease in sperm count, and sperm motility in a time- and dose-dependent manner. Seminal plasma SOD and sperm SOD activities were also decreased by all chromium (VI) treatments in duration- and dose-dependent manner. The activity of seminal and sperm CAT activity responded in the same way as SOD to the chromium (VI) treatment. The 100 p.p.m. chromium (VI)-treatment did not alter the seminal concentration of GSH. Nevertheless, higher doses of chromium (VI)-treatment significantly decreased the concentration of GSH in both seminal plasma and sperm. Meanwhile, the production of hydrogen peroxide was increased in all chromium (VI)-treated groups. The authors concluded that chromium (VI)-treatment could severely affect the fertility potential of males through the promotion of OS (Figure 3). The authors reported that sperm quality and antioxidant enzyme activities were restored to normal levels after 6 months of free-chromium exposure. The supplementation of the chromium (VI)-treated animals with vitamin C was able to prevent the deleterious effects of chromium (VI) on sperm quality and the activity of seminal and sperm antioxidant enzymes. These results suggest that the hazard effect of chromium (VI) can be naturally reverted over time in a chromium (VI)-free environment. Furthermore, the supplementation with vitamin C appears to prevent chromium (VI)-induced OS [107]. The conclusions obtained by Aruldhas M. and colleagues through their study in monkeys exposed to chromium (VI) were supported by several other animal studies [108,109,110,111,112]. So extensive studies are not possible to be performed in humans. While several studies reported that chromium (VI)-exposed male workers have a lower fertility potential [95,96,97], to our knowledge, no study has been done regarding the impact of chromium (VI) in the oxidative state of the human testis, seminal fluid, and spermatozoa. 

## 6. Conclusions

Industrialization has increased the exposure of all living creatures to a large variety of new compounds. Chromium (VI) is a powerful oxidant, common in the metal and paint industries. Due to its unstable electronic conformation, chromium (VI) has mutagenic and carcinogenic effects on cells, being a hazardous compound for all living beings. In the male reproductive system, chromium (VI) induces its deleterious effects through the promotion of OS. In the testis, it induces the complete disruption of spermatogenesis through the degradation of seminiferous tubules, chromatin fragmentation, mitochondrial dysregulation, and disruption of the blood–testis barrier [90]. Further, it decreases the activity of antioxidant enzymes and the concentration of non-enzymatic antioxidants, both in testis and seminal fluid. The severe oxidative state promoted by chromium (VI) ultimately leads to a decrease in sperm quality and male fertility potential [108,109,110,111,112]. Nevertheless, it appears that the harmful effects of chromium (VI) on the male reproductive system can be reversed either through time (in a chromium (VI)-free environment), or by supplementing males with an antioxidant compound, such as vitamin C [107]. This suggests that, although devastating, chromium (VI) does not promote irreversible damage to the reproductive tract. Individuals exposed to this hazard may be advised to use antioxidant supplements in order to prevent the loss of fertility associated with chromium (VI), increasing the chances of achieving a healthy pregnancy and offspring.

In contrast to chromium (VI), chromium (III) has been present in the list of essential elements for several decades. Its reported beneficial effects in glucose metabolism have turned the chromium picolinate, the most common commercialized form of chromium (III), into a popular diet supplement for people with metabolic disorders (such as diabetes and overweight/obesity) and athletes. Withal, the molecular mechanisms behind the action of chromium (III) are not completely understood, and reports of its efficacy are controversial. In the male reproductive system, high levels of chromium (III) appear to induce considerable damage to the testis, promoting atrophy of seminiferous tubules and compromising spermatogenesis [101]. However, these deleterious effects of chromium (III) on the testicular tissue have not been reported by all authors. Similarly, some authors reported a beneficial effect of chromium supplementation on sperm quality of breeding animals [105,106], while others found no differences [103,104]. The large heterogenicity of data probably results from the different methodologies used between studies. Not all studies used the same chromium (III) compounds: chromium (III) concentrations differ, and there is a large variety of animal models. It is also important to mention that the concentrations of chromium (III) used in these animal studies are usually higher than the dose of chromium (III) present in human dietary supplements. Regardless, the impact of chromium (III) on male fertility and overall human health needs to be further explored.
